# Determinants of modern family planning methods in Ethiopia: A community-based, cross-section mixed methods study

**DOI:** 10.1016/j.dialog.2022.100025

**Published:** 2022-06-23

**Authors:** Hocheol Lee, Eshetu Girma Kindane, Young Ah Doh, Eun Woo Nam

**Affiliations:** a Department of Health Administration, Yonsei University Graduate School, Wonju, Gangwon-do, Republic of Korea; b Department of Preventive Medicine, School of Public Health, Addis Ababa University, Ethiopia; c Korea International Cooperation Agency (KOICA), Gyungji-do, Republic of Korea; d Department of Health Administration, College of Health Science Yonsei University, Wonju, Gangwon-do, Republic of Korea

**Keywords:** Family planning methods, ETHIOPIA, Reproductive health, Population control, Birth-control

## Abstract

In 2019, Ethiopia had a total fertility rate of 4.2 births per woman with the rates varying significantly across regions. The Federal Ministry of Health of Ethiopia announced “Ethiopia FP 2020” to address the high fertility rate, aiming to reduce it to 3.0 by 2020. This study aimed to identify the determinants of the use of modern family planning services in the Amhara, Oromia, and Somali regions. A community-based, cross-sectional mixed methods study was conducted, using quantitative and qualitative surveys. The quantitative survey data were subjected to binary logistic regression analyses. Participants included over 4117 married men and women aged 15–65 years old. This study found that participants in Oromia were 8.673 times more likely to have modern family planning methods than those in Somali. Participants in Amhara were 5.183 times more likely to have modern family planning methods than their Somali counterparts. Women, married respondents, and recipients of media messages were more likely to have family planning experience. Family planning discussions with health extension workers and health professionals played a significant role in modern family planning. These findings show that establishing a family planning strategy that considers the sociocultural characteristics of each region help address regional contexts. Everyone in Somali—especially husbands and religious leaders—must be educated in family planning and funds be made available to deploy advanced measures for the same.

## 1 Introduction

The world's population has grown by 80 million people every year since 2000, exceeding 7.7 billion in 2019 [1]. Global population growth is expected to be concentrated in sub-Saharan Africa, which is home to the world's poorest countries, making population control in the region important. In particular, among the 20 countries with the highest population growth rates, 19 of them are located in sub-Saharan Africa [1]. The total fertility rate in developed countries, as defined by the Organisation for Economic Co-operation and Development (OECD), has decreased to 1.7, while in sub-Saharan Africa it has increased to 4.6 [1].

In 2017, to address the growing population problems in developing countries, the UK Department for International Development, the Bill & Melinda Gates Foundation, and United Nations Population Fund (UNFPA) invited recipient countries, international organizations, civilian groups, private organizations, and research institutes that were conducting family planning projects to hold an international conference and set common goals for worldwide population control [2,3]. In particular, they decided to support the right of an additional 120 million women of childbearing age in developing countries, including sub-Saharan Africa, to decide for themselves whether, when, and how many children to have. It was predicted that this would potentially prevent 100 million unwanted pregnancies, 50 million abortions, and 3 million maternal and child deaths [2]. According to the UNFPA report, family planning can reduce the total fertility rate, and improve the health levels of mothers and newborns. In addition, it is expected to improve the economic levels of families by increasing opportunities for women's social/economic activities and, in turn, lead to the economic development of countries [4].

In 2019, Ethiopia as the 12th most populous country in the world, had a total fertility rate of 4.2. It is expected to be the 8th most populous country in the world and 5th among developing countries by 2050 [1]. However, in Ethiopia, one in four people fail to meet their family planning needs [5]. Ethiopia faces polarization of the population between cities and local provinces, along with high total fertility rates and low family planning rates [6]. The difference in the total fertility rate between the Somali region, an area with high fertility, and Addis Ababa, an area with low fertility, in 2016 was 5.4 [5].

The Federal Ministry of Health (FMOH) of Ethiopia announced “Ethiopia FP 2020” to address the high fertility rate, with the goal of reducing the fertility rate to 3.0 by 2020 [7]. In March 2019, SDG Performance Funds, a meeting of the Ethiopian government, FMOH, and aid agencies in Ethiopia, discussed the budget allocation for family planning policies. The budget for Ethiopian family planning efforts has been a problem every year since the discussion started in 2012 [8]. The reason for this is that family planning resources were allocated in proportion to regions' populations. However, there was much disagreement as the project tried to curb an increase in the population. Accordingly, the meeting mentioned the importance of the basis for budget distribution by region and emphasized the need for research on family planning projects at the regional level [9,10].

This study aims to identify the determinants of the use of modern family planning methods in three regions of Ethiopia.

## 2 Materials and methods

### 2.1 Study design

This study used a community-based, cross-sectional mixed methods design. We selected the three largest out of the nine regions in Ethiopia: Amhara, Oromia, and Somali. The Amhara region is located in the northwestern part of Ethiopia, the Oromia region (which has the largest ethnolinguistic group in Ethiopia) is located in the central and western parts of the country [11], and the Somali region is located in the east and southeast.

The target group was married women and men living with a partner such as a husband, wife, or cohabitee in Amhara, Oromia, and Somali regions in Ethiopia.

### 2.2 Sample size and data collection

This study used two kinds of data: quantitative and qualitative. The sample size for the quantitative survey was determined using a double population proportion formula with the assumption of a 95% confidence level and 5% margin of error using G*Power software (version 3.0.10; Franz Faul, Kiel University, Kiel, Germany). The total sample size was estimated to be 2494 married couples, including a 10% non-response rate. Respondents in the three regions were selected using the multistage cluster sampling method. First, we selected towns in each region - six towns in Amhara, four in Oromia, and four in Somali. The six towns of Amhara region included Gozamin, Bahirdar, Dessie, Debrebirhan, Debremarkos, and Dessie; Oromia's four towns included Jima, Adama, Chiro, and Assela; and Somali's four towns included Jijiga, Gode, Siti, and Kebridehar. Second, we randomly selected a rural area and an urban area in each target town. Third, for homogeneity, we randomly selected 20–40 married couples per Kebele (the smallest administrative unit) in the Amhara, Oromia, and Somali regions.

The questionnaire used in this study was adapted from the USAID Demographic and Health Survey 2016 in Ethiopia [5]. This structured questionnaire was first developed in English by reviewing diverse studies on each region. Thereafter, the questionnaire was translated into three languages: Aaharic, Affan Oromo, and Somali by experts who fluently speak both English and these local languages. The data collection was conducted through face-to-face interviews by enumerators from May 10 to May 31, 2017. We used the Open Data Kit survey application on a tablet PC. We obtained data from 4688 respondents living in Amhara, Oromia, and Somali regions, with a response rate of 93.9%. Subsequently, we excluded 571 respondents' answers due to missing or censored data. The final sample for this study consisted of 1634 people from the Amhara region, 2383 from the Oromia region, and 100 from the Somali region.

We conducted qualitative in-depth interviews with the key informants living in the target regions. The questionnaire was developed based on the “Qualitative Study on Family Planning” by UNFPA.” Religious and community leaders helped us select the target respondents from whom we randomly selected 24 (8 per region) for key informant interviews.

### 2.3 Variables

The dependent variable was family planning practice experience. To measure this, we asked the respondents, “Have you (or your spouse) ever used any modern family planning methods (Pills, IUCD, Injectable, Implants, Emergency contraceptives, Foam, Condom, Vasectomy) to delay or stop a pregnancy?” A response of “Yes” was coded as “1” and other responses were coded as “0.” This question was taken from the Demographic and Health Survey 2016 (DHS 2016) by USAID [5].

The independent variables were socio-demographic characteristics including region, age, sex, religion, occupation, and marital status. We also selected family planning variables including a family planning discussion with partner (spouse, friend, family, health professional, health extension workers [HEW], religious leader, or community leader) and exposure to family planning messages.

The qualitative interview questionnaire had nine themes and 29 questions. We used seven themes: 1)fertility intention, 2)knowledge, perceptions & beliefs about contraceptive methods, 3) experience of contraceptive use, 4) reasons for discontinuing the use of the method, 5) reasons for non-use of contraceptive, 6) reasons for unwanted pregnancy and seeking induced abortion, and 7) future intention on contraceptive use and enabling factors.

### 2.4 Data analysis

The quantitative data were subjected to binary logistic regression analysis to identify the determinants of modern family planning methods across the three regions. This analysis was performed using SPSS Statistics 24.0.

The qualitative data were recorded using a digital recorder, and audio data were transcribed and then translated into English, saved as an MS Word file, and exported into Atlas-ti software for coding and categorization. Every item of the qualitative dataset was translated, and appropriate codes related to the evaluation objectives were assigned. Similar codes were grouped under the same category and a thematic content analysis was conducted. The themes were categorized into three types of reason: knowledge, interruption, and religion.

### 2.5 Ethical approval

Ethical approval was obtained from the Research Ethics Committee of the School of Public Health and the Institutional Review Board of the College of Health Science, Addis Ababa University (No. 032/17/SHP). Moreover, we obtained a support letter from the FMOH, regional health bureaus, and the study districts' health officers.

We obtained written informed consent from all participants for both the quantitative and qualitative surveys. We especially emphasized the respondents' right to refuse the interview. Couples were interviewed at the same time in separate places to avoid contaminating the data due to sensitive information being passed between spouses during the interviews.

## 3 Results

### 3.1 Characteristics of the study respondents

A total of 4117 married men and women (aged 15–65) participated. Geographically, participants were representative of Amhara (1634; 39.7%), Oromia (2383; 57.9%), and Somali (100; 2.4%). Table 1 depicts their characteristics by region. The mean age of the respondents was 32.2 ± 8.8 years and approximately half were male (1968; 47.8%). The identified religions of respondents were Muslim (1942; 47.2%) and Orthodox (1836; 44.6%). Most respondents were unemployed (2952; 71.7%).

**Table 1:** Respondent characteristics by region.

Variables	Total (*n* = 4117)		Amhara Population (*N* = 20,401,000)		Oromia Population (*N* = 33,692,000)		Somali Population (*N* = 6,453,000)	
			Respondents (*n* = 1634, 0.008%)		Respondents (*n* = 2383, 0.007%)		Respondents (*n* = 100, 0.002%)	
	N	%	N	%	N	%	N	%
FP^1^ experience								
No	1281	31.1	381	23.3	833	35.0	67	67.0
Yes	2836	68.9	1253	76.7	1550	65.0	33	33.0
Age								
15–19	129	3.1	37	2.3	85	3.6	7	7.0
20–29	1700	41.3	613	37.5	1058	44.4	29	29.0
30–39	1405	34.1	558	34.1	804	33.7	43	43.0
40–49	709	17.2	343	21.0	349	14.6	17	17.0
50–60	138	3.4	65	4.0	69	2.9	4	4.0
60–65	36	0.9	18	1.1	18	0.8	0	0
Sex								
Male	1968	47.8	805	49.3	1129	47.4	34	34.0
Female	2149	52.2	829	50.7	1254	52.6	66	66.0
Religion								
Protestant	132	7.9	50	3.1	274	11.5	0	0
Orthodox	1836	44.6	1100	67.3	728	30.5	8	8.0
Muslim	1942	47.2	481	29.4	1369	57.4	92	92.0
Catholic	15	0.4	3	0.2	12	0.5	0	0
Occupation								
Unemployed	2952	71.7	1174	71.8	138	72.9	40	40.0
Farmer	573	13.9	317	19.4	255	10.7	1	1.0
Trader	89	2.2	27	1.7	56	2.3	6	6.0
Houseworker	360	8.7	58	3.5	262	11.0	40	40.0
Daily worker	143	3.5	58	3.5	72	3.0	13	13.0
Marital status								
Married	3968	96.4	1582	96.8	2287	96.0	99	99.0
Cohabiting	149	3.6	52	3.2	96	4.0	1	1
Household monthly income								
≤ 500 ETB	120	2.9	50	3.1	70	2.9	0	2.9
501–1000 ETB	638	15.5	296	18.1	341	14.3	1	15.5
1001–1500 ETB	616	15.0	279	17.1	331	13.9	6	15.0
1501–2000 ETB	142	10.0	162	9.9	249	10.4	1	10.0
2001–2500 ETB	569	13.8	197	12.1	358	15.0	14	13.8
2501–3000 ETB	324	7.9	136	8.3	179	7.5	9	7.9
≥ 3000 ETB	1438	34.9	514	31.5	855	35.9	69	34.9
FP discussion with spouse								
No	515	12.5	205	12.5	264	11.1	46	46.0
Yes	3602	87.5	1429	87.5	2119	88.9	54	54.0
FP discussion with friend								
No	2886	70.1	1030	63.0	1784	74.9	72	72.0
Yes	1231	29.9	604	37.0	599	25.1	28	28.0
FP discussion with family								
No	4012	97.4	1559	95.4	2356	98.9	97	97.0
Yes	105	2.6	75	4.6	27	1.1	3	3.0
FP discussion with health professional								
No	3312	80.4	1349	82.6	1901	79.8	62	62.0
Yes	805	19.6	285	17.4	482	20.2	38	38.0
FP discussion with HEW^2^								
No	3767	91.5	1521	93.1	2176	91.3	70	70.0
Yes	350	8.5	113	6.9	207	8.7	30	30.0
FP discussion with religious leader								
No	4017	97.6	1564	95.7	2358	99.0	95	95.0
Yes	100	2.4	70	4.3	25	1.0	5	5.0
FP discussion with community leader								
No	4088	99.3	1618	99.0	2370	99.5	100	100
Yes	29	0.7	16	1.0	13	0.5	0	0
FP message exposure								
No	1876	45.6	541	33.1	1275	53.5	60	60.0
Yes	2241	54.4	1093	66.9	1108	46.5	40	40.0

1 FP = Family planning;

2 HEW = Health Extension Worker.

### 3.2 Quantitative analysis: Binary logistic regression

Respondents in Oromia were 8.673 times more likely to use modern family planning methods compared to those from the Somali region (AOR = 8.673, 95% CI: [5.160–14.581], *p* < .001). Moreover, those in Amhara were 5.183 times more likely to use modern family planning methods compared to those from the Somali region (AOR = 5.183, 95% CI: [3.147–8.538], *p* < .001) (Table 2).

**Table 2:** Binary logistic regression analysis of family planning (FP) experiences in the Amhara, Oromia, and Somali regions.

Variables	FP experience (%)		COR (95% CI)	AOR (95% CI)
	No	Yes		
Regions				
Somali	67(67.0)	33(33.3)	1	1
Oromia	833(35.0)	1550(65.0)	6.677 [4.333–10.288] ^⁎⁎⁎^	8.673 [5.160–14.581] ^⁎⁎⁎^
Amhara	381(23.3)	1253(76.7)	3.778 [2.469–5.780] ⁎⁎⁎	5.183 [3.147–8.538] ⁎⁎⁎
Age				
15–19	82(63.6)	47(36.4)	1	1
20–29	567(33.4)	1133(66.6)	3.486 [2.402–5.060] ⁎⁎⁎	3.481 [2.338–5.182] ⁎⁎⁎
30–39	314(22.3)	1091(77.7)	6.062 [4.146–8.864] ⁎⁎⁎	6.393 [4.226–9.673] ⁎⁎⁎
40–49	226(31.9)	483(68.1)	3.729 [2.520–5.517] ⁎⁎⁎	4.013 [2.594–6.206] ⁎⁎⁎
50–60	71(51.4)	67(48.6)	1.646 [1.009–2.687⁎]	2.053 [1.190–3.542] ⁎⁎
60–65	21(58.3)	15(41.7)	1.246 [0.587–2.647]	1.748 [0.772–3.961]
Sex				
Male	689(35.0)	1279(65.0)	1	1
Female	592(27.5)	1557(72.5)	1.417 [1.241–1.617] ⁎⁎⁎	1.764 [1.502–2.072] ⁎⁎⁎
Religion				
Protestant	98(30.2)	226(69.8)	1	1
Orthodox	460(25.1)	1376(74.9)	1.735 [0.479–6.283]	1.044 [0.267–4.083]
Muslim	720(37.1)	1222(62.9)	0.736 [0.571–0.949] ⁎	0.836 [0.636–1.100]
Catholic	3(20.0)	12(80.0)	1.297 [1.001–1.681] ⁎	1.164 [0.875–1.549]
Occupation				
Unemployed	903(30.6)	2049(69.4)	1	1
Farmer	188(32.8)	385(67.2)	0.792 [0.540–1.161]	0.965 [0.769–1.210]
Trader	22(24.7)	67(75.3)	0.715 [0.473–1.080]	1.677 [0.985–2.854]
Houseworker	131(36.4)	229(63.6)	1.063 [0.578–1.956]	0.746 [0.566–0.983] ⁎
Daily worker	37(25.9)	106(74.1)	0.610 [0.396–0.939] ⁎	1.531 [0.999–2.346] ⁎⁎⁎
Marital status				
Cohabiting	74(49.7)	75(50.3)	1	1
Married	1207(30.4)	2761(69.6)	2.287 [0.319–0.615] ⁎⁎⁎	2.277 [1.593–3.255] ⁎⁎⁎
Household monthly income				
≤ 500 ETB	36(30.0)	84(70.0)	1	1
501–1000 ETB	220(34.5)	418(65.5)	0.814 [0.533–1.243]	0.829 [0.528–1.300]
1001–1500 ETB	222(36.0)	394(64.0)	0.761 [0.498–1.162]	0.746 [0.475–1.173]
1501–2000 ETB	130(31.6)	282(68.4)	0.930 [0.597–1.447]	0.901 [0.562–1.446]
2001–2500 ETB	176(30.9)	393(69.1)	0.957 [0.623–1.470]	1.028 [0.649–1.628]
2501–3000 ETB	103(31.8)	221(68.2)	0.920 [0.583–1.450]	0.951 [0.583–1.551]
≥ 3000 ETB	394(27.4)	1044(72.6)	0.1.136 [0.756–1.706]	1.240 [0.800–1.922]
FP discussion with spouse				
No	225(49.5)	260(50.5)	1	1
Yes	1026(28.5)	2576(71.5)	2.462 [2.042–2.970] ⁎⁎⁎	2.426 [1.968–2.990] ⁎⁎⁎
FP discussion with friend				
No	976(33.8)	1910(66.2)	1	1
Yes	305(24.8)	926(75.2)	1.551 [1.334–1.804] ⁎⁎⁎	0.991 [0.830–1.182]
FP discussion with family				
No	1262(31.5)	2750(68.5)	1	1
Yes	19(18.1)	86(81.9)	2.077 [1.258–3.429] ⁎⁎	0.902 [0.515–1.582]
FP discussion with health professional				
No	1114(33.6)	2198(66.4)	1	1
Yes	167(20.7)	638(79.3)	1.936 [1.609–2.330]	1.488 [1.185–1.869] ⁎⁎⁎
FP discussion with HEW				
No	1224(32.5)	2543(67.5)	1	1
Yes	57(16.3)	293(83.7)	2.474 [1.848–3.313] ⁎⁎⁎	2.430 [1.698–3.476] ⁎⁎⁎
FP discussion with religious leader				
No	1268(31.6)	2749(68.4)	1	1
Yes	13(13.0)	87(87.0)	3.087 [1.717–5.550] ⁎⁎⁎	1.806 [0.922–3.539]
FP discussion with community leader				
No			1	1
Yes			1.187 [0.524–2.687]	0.427 [0.159–1.143]
FP message exposure through media				
No	641(34.2)	1235(65.8)	1	1
Yes	640(28.6)	1601(71.4)	1.298 [1.137–1.482] ⁎⁎⁎	1.210 [1.043–1.404] ⁎
Hosmer & Lemeshow				*P* = .803
Negelkerke R^2^				0.176

⁎ *P* < .05.

⁎⁎ *P* < .01.

⁎⁎⁎ *P* < .001

Based on gender, women were 1.764 times more likely to use modern family planning methods than men (AOR = 1.764, 95% CI: [1.502–2.072], *p* < .001).

Respondents whose occupation was housework were 0.746 times less likely to use modern family planning methods than the no-work group (AOR = 0.746, 95% CI: [0.566–0.983, *p* < .05]), or conversely those who were unemployed were 1.34 times more likely to use modern family planning methods compared to houseworkers. Daily workers were 1.531 times more likely to use modern family planning methods than respondents who were unemployed (AOR = 1.531, 95% CI: [0.999–2.346], *p* < .001).

Married respondents were 2.277 times more likely to use modern family planning methods than respondents who lived with a partner but were not married (AOR = 2.277, 95% CI: [1.593–3.255, p < .001]). Household monthly income was not a significant factor in modern family planning methods.

Respondents who discussed family planning with their spouse were 2.426 times more likely to use modern family planning methods than those who did not. Family planning discussions with HEWs (2.430 times) or a health professional (1.806 times) were significant factors in using modern family planning methods.

Respondents who received family planning messages through the media were 1.210 times more likely to use modern family planning methods than those who did not (AOR = 1.210, 95% CI: [1.043–1.404], *p* < .05).

### 3.3 Qualitative analysis

A total of 24 key informants participated in the study. Religious leaders, community leaders, HEWs, family planning (FP) service providers, and heads of health centers assisted with the qualitative interviews of this study.

Some of the points that emerged from the qualitative interview respondents belonging to different religions are listed below. The results showed that family planning practices could be divided into three focal points: knowledge, interruption, and religion.

#### 3.3.1 Knowledge

Some informants reported that they were aware of the importance of family planning for the health of both the mother and the newborn child. However, they said that opposition from their husband, spouse, or family stopped them from putting family planning strategies into practice.

*“Child-spacing benefits both the mother's and child's health, but often husbands don't like their wives when they have few children, and as a result they often marry a second wife to get many children. But I believe that child-spacing is good; for example, children who are born spaced are physically stronger than those who are born in succession” **[Somali, female, non-user**].*

On the other hand, many respondents did not use family planning because of incomplete or incorrect knowledge. Some respondents reported that family planning practices would cause a physical disease (such as skin disease, back pain, etc.), illness, or decreased sexual feeling.

*“… I am male, but I hear people saying once a woman uses this family planning, she will never give birth again as her reproductive organ will be closed. In cases where she lacks adequate food, the pills cause damage to her belly, skin discoloration (‘madiat’) on her face, etc. These are some of the doubts or fears in our community.”* ***[Oromia, male, non-user].***

*“… The drug results in weight gain and has a sensation of burning. It also makes the women aggressive and results in quarrels between husband and wife. It decreases love by reducing the sexual feelings of females; they also complain about pain during intercourse. I know many people who divorced because of this problem…”* ***[Amhara, male, Orthodox religious leader].***

#### 3.3.2 Interval in childbearing

Some respondents stopped using family planning services because they faced problems like pain, disease, headaches. The following quote is from an interview with a 30-year-old woman who used family planning for 7 years after the birth of her second child:

*“…After the second child, she again used an injectable for some time but stopped using it because she thought that it caused aggression, headaches, an internal burning sensation, and her menses did not flow…”* ***[Amhara, female, orthodox].***

There were no answers from the Oromia and Somali regions on this topic.

#### 3.3.3 Religion

Most respondents who did not practice family planning for religious reasons were Muslims. This is because the Muslim community considers family planning practices such as condoms, implants, UIDs, etc., as taboo under Sharia law. Therefore, Muslims have a low rate of practicing family planning. The following interviews were conducted with Muslims in the Amhara, Oromo, and Somali regions:

*“She aspires to have at least 10 children because she believes that having more children is more prestigious in the community and she will be respected more by her husband and his relatives. On the other hand, she believes that modern family planning methods are forbidden by Sharia and she has a plan to space her children using the calendar method and breast feeding, which are acceptable in Islam.”* ***[Somali, female, Muslim].***

*“….God said multiply and replenish the earth…children are gifts from God…when every child is born, he/she comes with his/her own fate/luck…”* ***[Amhara, female, Orthodox].***

*“I believe that the only one who makes every decision of the household including family size and family planning use [is the man] since he is entitled under Sharia law.”* ***[Oromia, male, Muslim].***

## 4 Discussion

In this study, the respondents' experience rate of modern family planning methods was 68.9% on average, with 75.7% in Amhara, 65.0% in Oromia, and 33.0% in Somali. The respondents discussed family planning with their spouses the most (87.5%), followed by friends (29.9%), health professionals (19.6%), HEWs (8.5%), religious leaders (2.4%), family (0.7%), and community leaders (0.7%). This concurs with the extant DHS data which found that people discussed family planning mostly with their spouses (72.8%) [5]. On the basis of region, family planning was discussed with spouses at the rate of 87.5% in Amhara, 88.9% in Oromia, and 54.0% in Somali region. According to DHS 2016, the rate in Amhara was 75.8%, 11.7% lower than in this study, and the rate in Oromia was 72.8%, or 16.1% lower than in this study. However, in Somali region (62.6%), the rate was 8.6% higher than in this study.

The quantitative analysis showed that the rate of usage of modern family planning methods was significantly different for each region in Ethiopia. The use of modern family planning methods in Oromia was 8.673 times higher compared to the Somali region (AOR = 8.673, 95% CI: [5.160–14.583], *p* < .001) and in Amhara was 5.183 times higher compared to the Somali region (AOR = 5.183, 95% CI: [3.147–8.538], *p* < .001). These results were associated with Somali's total fertility rate of 7.2, twice that of Amhara (3.7) and more than 1.5 times that of Oromia (5.4) [11]. According to the quantitative and qualitative analyses, because 98.7% of the local residents in the Somali region followed Islam and family planning (contraception) is prohibited under Sharia law, it is rarely used there. In particular, the use of condoms, implants, pills, and IUDs for family planning is prohibited, while only the calendar method and breastfeeding is permitted. In fact, the Quran clearly states, “Bring a lot of children for the prosperity of Islam”, so there are limitations in implementing family planning policies for Muslims [12,13,14].

According to a study by Tigabu [13] on religious views on contraception in western Ethiopia, there is a belief that if a female Ethiopian Muslim dies with an implant, a female contraceptive device, in her body, her soul cannot go to heaven.

On the other hand, there were some opinions that ran contrary to our qualitative findings and the findings of previous studies. One Muslim woman responded that family planning was necessary because she perceived that her children were born healthy because of family planning, which controlled the age gap of her children. This demonstrates that, even though women themselves recognize the need for family planning, they fail to implement it for religious reasons. There is a 3.4% level of use of family planning in Somali region, where 98.7% of the population is Muslim, while 79.0% of women are aware of the importance of family planning [5]. There are, thus, many women who cannot practice family planning (contraception) for religious and doctrinal reasons, despite their wishes. Traditionally, Muslim husbands have all the decision-making power. A previous study on the use of condoms in Ethiopia found that because family planning in Muslim families was decided by the husband, women faced many limitations in using/practicing modern family planning methods on their own [15,16].

In this study, the proportion of women who decided on family planning after consultation with their husband was 54.0% in the Somali region, lower than in Amhara (87.5%) and Oromia (88.9%). Therefore, in the Somali region, it is important to educate religious leaders and heads of the family (e.g., husbands) about the importance of family planning (contraception) to increase the rate of family planning practice, thereby lowering the total fertility rate. In fact, after conducting educational sessions on family planning with Muslim religious leaders and men in three groups, it was found that their perceptions changed, which lowered the fertility rate and gestational disease rates [17,18].

In Ethiopia, family planning projects such as education for health professionals, HEWs, and religious leaders; media education; and the distribution of brochures are carried out by various organizations including international organizations and NGOs, research institutes, and civic groups [8,[19], [20], [21]]. Among them, the Small, Happy, and Prosperous family in Ethiopia, conducted by the Korea International Cooperation Agency, was identified as the most effective intervention for delivering family planning educational messages through the media [22,23]. However, the Somali region has the lowest message exposure (radio: 4.8%, TV: 6.6%), so there are limitations in delivering family planning education content through the media [5]. Therefore, family planning education in the Somali region should involve 1) supplying equipment to send messages (to improve message exposure), and 2) providing direct education to religious leaders, husbands, and medical professionals. The details are as follows.

First, providing equipment (radios, TVs) to listen to messages will help improve message exposure in the Somali region and increase the effectiveness of education delivered through the media. In this study, the number of people with experiences of receiving family planning messages through the media (exposure experience) was 1.12 times higher (AOR = 1.120, 95% CI: [1.043–1.404], *p* < .05) than the number of people with no experience of using family planning services. In particular, the transmission of messages has proved to be an effective and widely used intervention in previous studies [23,24].

Second, education to increase awareness of family planning is necessary for family decision-makers and certain influential groups such as husbands, health professionals, and HEWs. In this study, the use of modern family planning methods depended on with whom these discussions were held. Those that discussed whether to use modern family planning methods with HEWs, which had the highest influence, were 2.430 times (AOR = 2.430, 95% CI: [1.698–3.476], *p* < .001) more likely to use the services compared to those that did not, and this result was statistically significant. This is because Ethiopian females were worried about getting scolded by their husbands. They therefore relied on and consulted the most with HEWs who were of the same gender and not a family member [25]. The second most influential group to discuss using modern family planning methods were spouses; the use of modern family planning methods after a discussion with a spouse increased by 2.426 times (AOR = 2.426, 95% CI: [1.968–2.990], *p* < .0001) as compared to the absence of a discussion. The third most influential group was health professionals, where the use of family planning services was 1.448 times higher (AOR = 1.488, 95% CI: [1.185–1.869], *p* < .001) after a discussion with them.

On the other hand, discussions with friends, religious leaders, and community leaders were not significantly related to the use of modern family planning methods. Usage of these services increased when consulting with a decision-maker or influential health professional, but in some cases, false information prevented people from using them. According to the interviews in the qualitative survey, a male respondent heard from his neighbors that using modern family planning methods would block the female reproductive system and prevent his wife from future childbirth and he cited this as the reason for not using it. This shows that incorrect knowledge among major decision-makers such as husbands can act as a barrier to family planning.

One limitation of this study were the discrepancies in the responses to the qualitative and quantitative surveys. The respondents of the qualitative survey were interviewed regardless of whether they had participated in the quantitative survey. To minimize errors in the quantitative and qualitative analytical results, there needs to be consistency in the respondents [26]. However, as we could not conduct corresponding qualitative surveys for all 4117 respondents of the quantitative survey, the qualitative respondents were selected via conditional extraction. Moreover, the Somali region sample had only 100 respondents, because Muslim women avoided talking with outsiders and did not want to participate in the survey as it was about family planning. In future studies, it is necessary to minimize the errors in the analysis by equalizing the respondents in the qualitative and quantitative surveys.

Moreover, this study was conducted in three out of nine regions of Ethiopia: Amhara, Oromia, and Somali. In future studies, it is necessary to minimize errors by conducting surveys in all nine regions.

## 5 Conclusion

This study aimed to identify the determinants of the use of modern family planning methods in Ethiopia using a mixed method research design based on both quantitative and qualitative surveys. This study was conducted in the three largest regions of Ethiopia: Amhara, Oromia, and Somali.

Compared to the Somali region, the use of modern family planning methods was significantly higher in the Amhara region (5.183 times higher) and the Oromia region (8.673 times higher). The reason, confirmed through the qualitative research, was that 98.7% of those in the Somali region were Muslims and there were limits in using modern family planning methods due to the absolute right of husbands to decide on family planning, the prevailing religious doctrine, and a lack of knowledge. In particular, although the results showed that people were aware of the importance of family planning, for religious reasons, it was not possible to use it.

In addition, the use of modern family planning methods differed significantly depending on the person consulted. When participants consulted with their husband on whether to use modern family planning methods, usage increased by 2.426 times (a significant difference) compared to those that did not. When they consulted with a religious leader, usage increased by 1.806 times (not statistically significant). In addition, those that listened to a family planning message through the media had an increased usage of modern family planning methods by 1.210 times (a significant difference).

Thus, it is necessary for Ethiopia to establish a family strategy in consideration of the cultural and social characteristics of each region. In particular, the Somali region needs to educate husbands and religious leaders about the importance of family planning. As there exists a shortage of media equipment, supplying it or organizing a common radio/TV spot is necessary to enable their access to family planning messages.

## Ethics approval and consent to participate

Ethical approval was obtained from the Research Ethics Committee of the School of Public Health and the Institutional Review Board of the College of Health Science, Addis Ababa University (No. 032/17/SHP). We obtained written informed consent from all participants for both quantitative and qualitative surveys. In particular, this study includes women aged 15–19 who are mothers with children. In the case of women aged 15–19, we received consent from their parents. Because the questionnaire included family planning (contraception), sexual issues, sexual knowledge, intercourse experience, etc., parents' consent was obtained because the content may be inappropriate for women under 19 years.

We especially emphasized the respondents' right to refuse the interview. Couples were interviewed at the same time in separate places to avoid contaminating the data due to sensitive information being passed between spouses during the interviews.

## Consent for publication

Not applicable.

## Availability of data and materials

The data used for the current study is available from the corresponding author on reasonable request.

## Funding

This research was funded by 10.13039/501100003605Korea International Cooperation Agency (KOICA), grant number P2019-00160-1.

## CRediT authorship contribution statement

**Hocheol Lee:** Conceptualization, Formal analysis, Methodology, Writing – original draft. **Eshetu Girma Kindane:** Formal analysis, Writing – review & editing. **Young Ah. Doh:** Funding acquisition. **Eun Woo Nam:** Conceptualization, Methodology, Writing – review & editing.

## Declaration of Competing Interest

The authors declare no conflict of interest.
